# More Social needed by the Sciences

**DOI:** 10.1038/s44319-025-00615-1

**Published:** 2025-10-30

**Authors:** Frank Gannon

**Affiliations:** QIMR Berghofer Medical Research Institute, Brisbane, QLD Australia

**Keywords:** Pharmacology & Drug Discovery, Science Policy & Publishing

## Abstract

All too often, scientific facts and nuances get lost or distorted in communication. Involving social scientists in research projects and the messaging of the outcomes could improve  interactions with  the public.

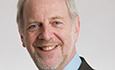

Many years ago, in the 1980s, my lab was visited by a TV crew because they had heard that we had discovered something that could develop into a cure for cancer. The mutation that we found was indeed interesting, and I presented a cautious view of the potential long-term impact on cancer therapy. The interviewer stopped the session and said, “Either you say you have a cure for cancer or there is no story”. On the rerun, having put in a few ifs and buts, I ended by saying it could be a cure for cancer. On the TV news, this was the only phrase they used. Nuance was hard to convey back then and still is today. Research scientists have not been trained in communication and how to get their messages across to people who are not familiar with the concepts and the details. And scientists who are successfully explaining science to the laypeople have been pilloried for wasting their time by communicating complex messages to the public (Caplan, 2025).

How often have we heard the phrase “the science says” as some new truth is promoted? However, it does not take long before a rare side effect appears. Amplified by social media, the overwhelmingly positive balance between population benefit and individual damage is forgotten. This has been the case for Covid vaccinations, where politics and health became intertwined, and it did not take long for loud voices to trumpet views that convinced some of their followers to see the vaccine as a threat rather than a redemption. Or falsified data is published, and even if it is discredited, the “result” is used for decades to promote a particular belief, as happened with the long-debunked claim of a link between vaccination and autism. No matter how solid the positive evidence from “the science”, the potential for negative outcomes gets equal airtime. When someone a community trusts—such as a politician they vote for—says that they are convinced that a treatment is bad, they are listened to rather than the assertions of good from a white coat from a different world.

Covid was not a unique example of the problem of objective scientific data being ignored in favour of subjective preferences. For years, we have known that too much salt is bad, too much sugar is bad, too much sun is bad, too much alcohol is bad, being obese is bad, and collectively that over one third of diseases are preventable. Yet we do not make the necessary adjustments to our lifestyle. It is only when the consequences of our choices seem to be linked to a personal illness that we start to adjust—sometimes too late. It is not sufficient to end a presentation with what “the science” says; the public has to receive the message in a way that impacts them as individuals.

While researchers do a great job in understanding the causes of a disease, identifying a possible cure, and testing its effectiveness, it is clearly not sufficient. The weak link, I suggest, is that there is an internal conundrum in our messaging. We present facts that have been proven, but the scientific process means that these facts will and should be challenged over time by new information and approaches (Jacobs, 2025). “Truth” can be ephemeral when dealing with the complexities of biology. The caveats that we put in our publications are not understood or are lost when the message gets distilled into “this is a cure for cancer”. Getting the message to our colleagues is easy, but the general public is a different group that we do not talk to sufficiently, using messaging that will resonate. Joel Mokyr, the recent winner of the Nobel Prize in Economic Sciences, traced the pre-requisites of sustained growth through innovation and emphasised the importance of society being open to new ideas and change. That requires effective communication from those who make those discoveries.

Admittedly, progress is being made in broadening scientists’ engagement beyond the research world. Increasingly, patient groups and other communities are part of discussions on where research should focus to ensure that the outcome will be beneficial for them. In the process, the scientists learn how to better communicate with them. However, once the clinical trial is completed, the authenticity of the message can be warped. The biotech company releasing the news has to cater to its investors and the pharma company to its shareholders as they extoll the new product. The patients are again on the receiving end of communication of the medical system, and again, the language is wrapped in a message, “I know what you need”. Outside the consulting rooms, a different communication dominates. It is full of anecdotes, exceptions, and exaggerations. With a few words, amplified by social media and ideological biases, it is possible to convince many that vaccines are evil, that an everyday drug to overcome a minor health problem can damage the unborn child, and that a quack solution to a complex disease is all that is needed for a cure.

More engagement by social scientists is therefore essential. They are the ones who understand how society works and how messages are best presented. They can prepare communication strategies to ensure that sane advice is presented to and accepted by the public. They can minimise the risk that the self-interest of the scientists and the companies distorts the message.

When the scientific community considered the establishment of the European Research Council (ERC), the social sciences were merely tagged on to the discussions on the ERC’s potential scope. It was always “**and** the social sciences” and nearly all sides saw “them” as distinct from biology, chemistry, or physics. Instead, it could be fruitful if social scientists were integral members of any major grant award. In their application for funds, the wet-lab scientists should have to include an outline of the communication strategy along with a proposed pathway of how to move their product into the clinic for the benefit of the patients.

The reverse mixing of cultures and skills also should happen more frequently. Authors and playwrights could be offered the opportunity to meet and mingle with researchers and clinicians to get inspiration from and background on scientific and health topics. Many great works of literature, theatre, and cinema have covered the early HIV/AIDS era. Are there opportunities to add to the storytelling by presenting contemporary and forward-looking research topics to the public? Getting messages into the mainstream this way would be more effective than trying to react to misinformation when it all seems to be a whitewashing of a wall to which the mud has already stuck. Science is a powerful positive force, but it is too often a challenge to explain it to the people. Adding social sciences to the mixture would be an essential method of converting the good into the impactful.

## Supplementary information

Peer Review File
