# Supplementary material for: More Social needed by the Sciences
Source: EMBO Rep. 2025 Oct 30;26(23):5637–8. doi: 10.1038/s44319-025-00615-1 (PMC12678395; doi:10.1038/s44319-025-00615-1)
Supplement: Supplementary file 1 — Peer Review File [file 44319_2025_615_MOESM1_ESM.pdf]

EMBO reports

More Social needed by the Sciences

Frank Gannon

Corresponding author(s): Frank Gannon (FGannon@qimrberghofer.edu.au)

**Review Timeline:**	Submission Date:	15th Oct 25
	Accepted:	16th Oct 25

Editor: Holger Breithaupt

Transaction Report: This article type is not peer reviewed.
